# Near infrared spectroscopy for body fat sensing in neonates: quantitative analysis by GAMOS simulations

**DOI:** 10.1186/s12938-016-0310-y

**Published:** 2017-01-11

**Authors:** Fatin Hamimi Mustafa, Peter W. Jones, Alistair L. McEwan

**Affiliations:** School of Electrical and Information Engineering, Faculty of Engineering, University of Sydney, New South Wales, Australia

**Keywords:** Near-infrared spectroscopy, Body fat sensing, Fat thickness, GAMOS simulation, Neonates, Under-nutrition detection

## Abstract

**Background:**

Under-nutrition in neonates is closely linked to low body fat percentage. Undernourished neonates are exposed to immediate mortality as well as unwanted health impacts in their later life including obesity and hypertension. One potential low cost approach for obtaining direct measurements of body fat is near-infrared (NIR) interactance. The aims of this study were to model the effect of varying volume fractions of melanin and water in skin over NIR spectra, and to define sensitivity of NIR reflection on changes of thickness of subcutaneous fat. GAMOS simulations were used to develop two single fat layer models and four complete skin models over a range of skin colour (only for four skin models) and hydration within a spectrum of 800–1100 nm. The thickness of the subcutaneous fat was set from 1 to 15 mm in 1 mm intervals in each model.

**Results:**

Varying volume fractions of water in skin resulted minimal changes of NIR intensity at ranges of wavelengths from 890 to 940 nm and from 1010 to 1100 nm. Variation of the melanin volume in skin meanwhile was found to strongly influence the NIR intensity and sensitivity. The NIR sensitivities and NIR intensity over thickness of fat decreased from the Caucasian skin to African skin throughout the range of wavelengths. For the relationship between the NIR reflection and the thickness of subcutaneous fat, logarithmic relationship was obtained.

**Conclusions:**

The minimal changes of NIR intensity values at wavelengths within the ranges from 890 to 940 nm and from 1010 to 1100 nm to variation of volume fractions of water suggests that wavelengths within those two ranges are considered for use in measurement of body fat to solve the variation of hydration in neonates. The stronger influence of skin colour on NIR shows that the melanin effect needs to be corrected by an independent measurement or by a modeling approach. The logarithmic response obtained with higher sensitivity at the lower range of thickness of fat suggests that implementation of NIRS may be suited for detecting under-nutrition and monitoring nutritional interventions for malnutrition in neonates in resource-constrained communities.

**Electronic supplementary material:**

The online version of this article (doi:10.1186/s12938-016-0310-y) contains supplementary material, which is available to authorized users.

## Background

The World Health Organization (WHO) recorded about 104 million children were undernourished in 2010 with most being neonates in developing countries [1]. Undernourished neonates were generally small and had low body fat. They need sufficient amount of fat in their body because the fat provides energy to fight infections, resistance to high and low temperature, and protection against hypoglycemia and hypothermia [2]. Several technologies for measuring body fat include computer tomography, ultrasound imaging, dual-energy X-ray absorptiometry and air displacement plethysmography. These are expensive and need trained operators [3]. Skinfold thickness is low-cost but suffers from observer variability [4], while deuterium dilution measurements incur a delay for sample processing [5]. Additional file 1: Table S1 shows the comparison of body composition methods using a Figure of Merit (FOM) equation according to estimated cost (including equipment set-up), estimated measurement time, requirement for skilled operators, noninvasiveness, mobility, and safety [3, 6]. Also included the primary measurements of each method (see Additional file 1).

Near infrared spectroscopy (NIRS) method has a great potential for undernutrition monitoring because it is safe, fast, non-invasive, mobile, relatively low-cost, and can be directly connected to portable computing devices, which makes this method is feasible to be applied easily on neonates in low-middle income settings. The term ‘body fat measurements using NIRS’ is related to measurement of the amount of adipose tissue underneath skin using NIR spectroscopy. The NIRS for measuring body fat has also been applied for other purposes such as in dietary monitoring [7–12], and also in liposuction surgery, where the condition of adipose tissue is checked pre-surgery and post-surgery [11, 13]. In muscle oxygenation measurements using NIRS, the effect of fat thickness from NIRS measurements is corrected using developed models [14, 15].

While NIRS provides several advantages, the limitations of using the NIR spectrum however include its sensitivity to hydration and skin colour [16]. This motivated the first aim of this study, which was to study quantitatively the effect of different skin colours and different hydration on NIRS measurements of the skin. We develop two single fat layer models and four skin models using simulations having varied volume fractions of melanin (skin colour), *V*_*melanin*_ and volume fractions of water (hydration), *V*_*water*_. Note that the variation of *V*_*melanin*_ is only for the skin models because melanin only presents in the epidermal layer of the skin. The single fat layer models at varied *V*_*water*_ are developed in order to get picture of basic response of the interest layer.

The second aim of our study was to define the sensitivity and the relationship between the reflected NIR intensity and the thickness of fat. We set a range of fat thickness in the two developed single fat layer models and in the four developed skin models from 1 to 15 mm in 1 mm intervals in the simulation. Past studies by simulation and/or phantom experiment showed inconsistency between the relationships proposed by the various studies, which exhibited either a logarithmic, an exponential or a peak response of NIR reflection with the increase in thickness of subcutaneous fat [7–14]. Additional file 2: Table S2 summarises a literature review of fat measurement using NIRS by simulation and phantom experiment (see Additional file 2).

We improve our earlier study [12] in several aspects including assignment of optical properties. We exploit Meglinski’s equation model by changing values of *V*_*water*_ and *V*_*melanin*_ in the equation to define new absorption values suiting the two developed single fat layer models and the four developed skin models. Note that we previously used absorption coefficient, *μ*_*a*_ data directly from Simpson et al. [17] and then implemented it into our previous developed equation model [12]. In this paper, we also consider changes values of refractive indices, *n*, and reduced scattering coefficient, $\mu_{s}^{{{\prime}}}$ due to changes of melanin and water in the skin tissue.

Our study is also different from [12] in term of source-detector configuration. In the simulation, we follow the real source-detector device specifications and parameters. We introduce an arrangement of the source and the detector having +45° and −45° angles to the skin surface respectively (i.e. a 90° included angle between the axes of the source and detector) with 10 mm separation. The position angle of the source-detector is different from that commonly presented in the literature, where both the source and detector were aligned at +90° to the skin surface (i.e. the axes of the source and detector were parallel) (see Additional file 2: Table S2) [7–14]. We select 45° angles because we have shown in a previous study that performance in terms of sensitivity shown by 45° angles was higher than 90° angles in measuring body fat using NIRS [18].

We perform the simulation using Geant4-based Architecture for Medicine Oriented Simulation (GAMOS), an open-source software package that applies the Monte Carlo simulation method. GAMOS was first developed in 2006 for medical physics applications and provides easy design simulations without requiring C++ coding. A tissue optics plug-in interfaced with GAMOS was then introduced in 2013 [19]. In a validation study with accepted standards within the biomedical optics community between GAMOS and other simulation methods such as Monte Carlo Multilayer (MCML), GAMOS performed with the lowest error of total diffuse reflectance, *R*_*d*_ [19].

## Methods

**Fig. 1:**
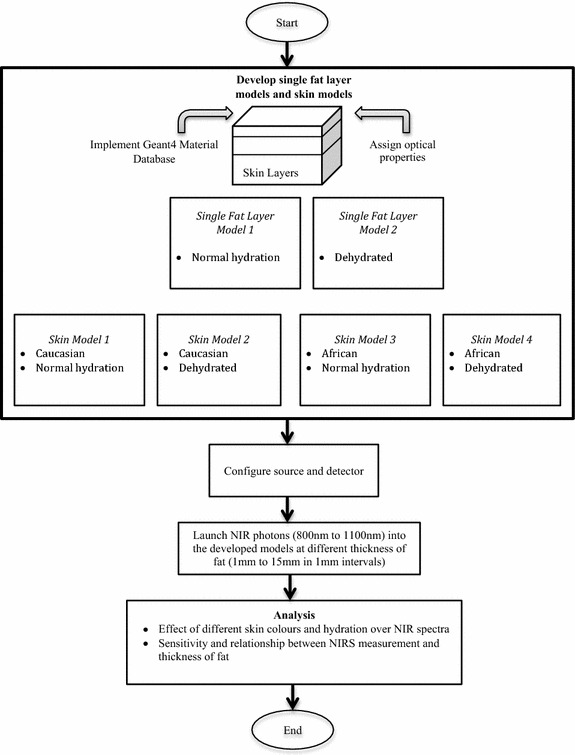
Flow-chart of methodologies and steps involved in GAMOS simulation for body fat sensing using NIRS

**Fig. 2:**
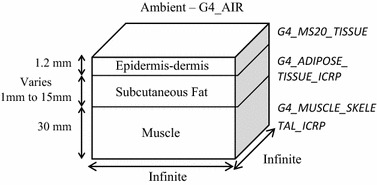
Implementation of GMD and assignment of thickness on epidermis–dermis, subcutaneous fat and muscle layer

Figure 1 illustrates a flow chart of methodologies and steps involved in setting up GAMOS simulation for body fat sensing using NIRS. Two (2) single fat layer models (having dimensions infinite × *variable fat thickness* × infinite) at different *V*_*water*_ were developed. The single fat model 1 was the single fat layer having normal hydration while dehydrated single fat layer was for the single fat layer model 2. Four (4) skin models were also developed at different *V*_*water*_ and *V*_*melanin*_ each consisting of an upper epidermis-dermis, a middle subcutaneous fat layer and a lower muscle layer. The skin model 1 and the skin model 2 were Caucasian skin models (lower *V*_*melanin*_) with normal hydration (higher *V*_*water*_) and dehydrated (lower *V*_*water*_) respectively. The skin model 3 was the African skin (higher *V*_*melanin*_) having normal hydration (higher *V*_*water*_) while the dehydrated African skin (higher *V*_*melanin*_ and lower *V*_*water*_) was used in the skin model 4. In each model, the thickness of the fat layer was varied from 1 mm to 15 mm in 1 mm intervals. Epidermis and dermis were combined into one single layer following models validated in the literature [17]. For mimicking real tissue, the models were implemented with the Geant4 Material Database (GMD) function invoking suitable materials. Figure 2 shows the implementation of GMD on a developed skin model as well as the assignment of thickness to each layer. The thicknesses follow reported values from real neonatal skin [20].

By implementing GMD, materials or mixture properties of mass per mole, density state and pressure were composed by a number of elements. For instance, material of *G4_ADIPOSE_TISSUE_ICRP* was made from combinations of hydrogen, carbon, nitrogen, oxygen, sodium, sulfur and chlorine elements forming total density of 0.95 g/cm^3^. Properties and compositions of *G4_AIR* material and *G4_MS20_TISSUE* material refer to the National Institute Standard and Technology (NIST) standard, while *G4_ADIPOSE_TISSUE_ICRP* and *G4_MUSCLE_SKELETAL_ICRP* refer to the International Commission on Radiological Protection (ICRP) standard [21].

Optical properties as a function of wavelength in terms of absorption coefficient, *μ*_*a*_, refractive indices, *n*, reduced scattering coefficient, $\mu_{s}^{{{\prime}}}$ and anisotropy, *g* were then assigned to each layer of the developed models. The values of optical properties were defined based on changes of *V*_*water*_ and *V*_*melanin*_ in adult tissue. Data from adults were used due to an absence of published data for neonates at NIR wavelengths. A simplified form of Meglinski’s equation model for determining *μ*_*a*_ of the tissue layer was [22–24];

1 $$\begin{aligned} \mu_{a}^{layer} \left( \lambda \right) = & \left[ {V_{fat} \mu_{a}^{fat} (\lambda )} \right] & + \left[ {V_{water} \mu_{a}^{water} (\lambda )} \right] + \left[ {V_{blood} \mu_{a}^{blood} (\lambda )} \right] + \left[ {V_{melanin} \mu_{a}^{melanin} (\lambda )} \right] \\ + \left[ {\mu_{a}^{{intrinsic}} \left( \lambda \right)\left( {1 - V_{fat} - V_{water} - V_{blood} - V_{melanin} } \right)} \right] \\ \end{aligned}$$

where, *λ* is the wavelength in *nm* while *μ*_*a*_^*layer*^(*λ*) is the absorption coefficient of the tissue layer (either epidermis-dermis, subcutaneous fat or muscle). *V*_*blood*_ is the volume fraction of blood while *V*_*fat*_ is the volume fraction of fat. *μ*_*a*_^*blood*^(*λ*)*, μ*_*a*_^*water*^(*λ*), *μ*_*a*_^*melanin*^(*λ*) and *μ*_*a*_^*fat*^(*λ*) indicate the absorption coefficients spectra of the blood, water, melanin and fat constituents respectively while *μ*_*a*_^*intrinsic*^(*λ*) is the absorption coefficient of skin free of any absorber that was expressed by [25];

2 $$\mu_{a} \left( \lambda \right)^{intrinsic} = 7.84 \times 10^{7} \lambda^{ - 3.255}$$

**Fig. 3:**
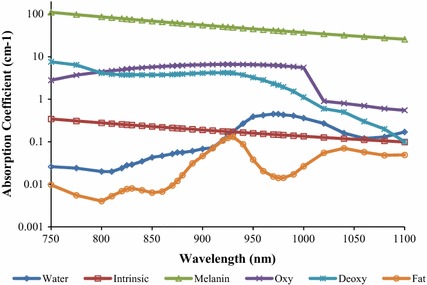
Absorption coefficient spectra of primary absorbers in the skin tissue. Absorption coefficient spectra of water, *μ*_*a*_^*water*^(*λ*), melanin, *μ*_*a*_^*melanin*^(*λ*), fat, *μ*_*a*_^*fat*^(*λ*), deoxy- hemoglobin, *μ*_*a*_^*Hb*^(*λ*), oxy-hemoglobin, *μ*_*a*_^*Hb02*^(*λ*), and intrinsic, *μ*_*a*_^*intrinsic*^(*λ*)

The absorption coefficient spectra of the blood, *μ*_*a*_^*blood*^(*λ*) in Eq. (1) was defined as [22];

3 $$\mu_{a}^{blood} \left( \lambda \right) = \left[ {(1 - V_{oxy} )V_{hemoglobin} \,\mu_{a}^{Hb} (\lambda )] + [V_{oxy} V_{hemoglobin} \,\mu_{a}^{Hb02} (\lambda )]} \right]$$

where *V*_*hemoglobin*_ and *V*_*oxy*_ are the volume fractions of hemoglobin and oxygen saturation, which were assigned the values of 0.6 and 0.1 respectively [23]. *μ*_*a*_^*Hb*^(*λ*) is the absorption coefficient spectra of the deoxy-hemoglobin while *μ*_*a*_^*Hb02*^(*λ*) is the absorption coefficient spectra of the oxy-hemoglobin. The melanin, hemoglobin and small-scale tissues were assumed be evenly distributed in the epidermis-dermis. Figure 3 illustrates the spectra for *μ*_*a*_^*Hb*^(*λ*)*, μ*_*a*_^*Hb02*^(*λ*), *μ*_*a*_^*water*^(*λ*), *μ*_*a*_^*melanin*^(*λ*),* μ*_*a*_^intrinsic^(*λ*) and *μ*_*a*_^*fat*^(*λ*), which are also known as the primary absorbers in skin tissues. The value of *μ*_*a*_^*water*^(*λ*) was obtained from Hale et al. [26], the value of *μ*_*a*_^*fat*^(*λ*) was defined from Veen et al. [27], while the *μ*_*a*_^*Hb*^(*λ*), *μ*_*a*_^*Hb02*^(*λ*) and *μ*_*a*_^*melanin*^(*λ*) values were referred from Jacques et al. [16]. Those values and information of the *μ*_*a*_(*λ*) (water, fat, hemoglobin and melanin) can be obtained from the Oregon Medical Laser Center, *omlc* website [28].

**Fig. 4:**
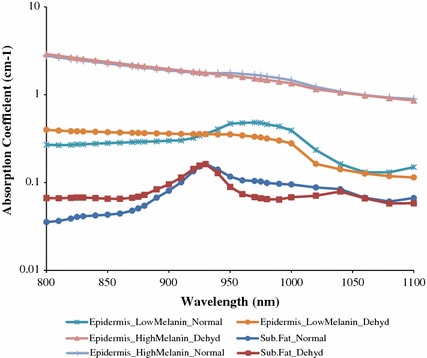
Absorption coefficient spectra from the values obtained using Eq. (1). Absorption coefficient spectra of epidermis–dermis, $\mu_{a}^{epidermis}$ ($\lambda$) at varying combinations of *V*_*water*_ (normal or dehydrated (Dehyd)) and *V*_*melanin*_ (low or high) as well as absorption coefficient spectra of subcutaneous fat, $\mu_{a}^{subcutaneous}$ ($\lambda$) at varying combinations of *V*_*water*_ (normal or dehydrated)

The values of the volume fractions in Eq. (1) were referred to the portion of constituents in the tissue layers given by the literature [29–34]. Table 1 shows the values of *V*_*blood*_ *, V*_*water*_ *, V*_*melanin*_, and *V*_*fat*_ in epidermis–dermis, subcutaneous fat layer and muscle layer. *V*_*intrinsic*_ contains constituents having a lower absorption effect in skin such as potassium and sodium [30–32]. Substituting of the *μ*_*a*_ values of constituents (Fig. 3) and their corresponding volume fractions (Table 1) into Eq. (1), absorption coefficients spectra of epidermis-dermis, *μ*_*a*_^*epidermis*^ (*λ*), subcutaneous fat layer, *μ*_*a*_^*subcutaneous*^ (*λ*) and muscle layer, *μ*_*a*_^*muscle*^ (*λ*) were obtained at varying *V*_*water*_ and *V*_*melanin*_ in Fig. 4. The *μ*_*a*_^*muscle*^ (*λ*) is not shown in Fig. 4 because it was not involved in studying the effect of water and melanin. The graphs in Fig. 4 are plotted from the values obtained using Eq. (1).

**Table 1:** Volume fractions of *V*_*blood*_, *V*_*melanin*_, *V*_*fat*_, *V*_*water*_, and *V*_*intrinsic*_ in skin layers

Layers	*V*_*blood*_	*V*_*melanin*_	*V*_*fat*_	*V*_*wate*r_ (normal)	V_water_ (dehydrated)	V_intrinsic_
Epidermis dermis 1 Caucasian	0.3	0.0010	0	0.6	*0.1*	1.0 − (*V*_*blood*_ + *V*_*melanin*_ +* V*_*water*_)
Epidermis dermis 2 African	0.3	0.030	0	0.6	*0.1*	1.0 − (*V*_*blood*_ + *V*_*melanin*_ +* V*_*water*_)
Subcutaneous fat	0.05	0	0.79	0.14	*0.02*	1.0 − (*V*_*blood*_ +* V*_*water*_ + *V*_*fat*_)
Muscle	0.20	0	0	0.70	–	1.0−(*V*_*blood*_ +* V*_*water*_)

The refractive index, *n* has a direct relationship with the reduced scattering coefficient $\mu_{s}^{{{\prime}}}$[33], therefore if one of them changes due to vary melanin or water in the skin tissue, the other parameter was assumed to change equally. The anisotropy, *g* was set as 0 in all conditions (varied *V*_*water*_ and *V*_*melanin*_) presuming the constant variable of (*1 *− *g*) from an equation of $\mu_{s}^{{{\prime}}}$ = (*1* − *g*) *μ*_*s*_, where the $\mu_{s}^{{{\prime}}}$ proportions to the *μ*_*s*_, the scattering coefficient. The value of the *g* (*g* = *0*) was chosen because GAMOS has not yet offered GPU-based acceleration or mesh-based grid generation particularly for quantitative analysis [19]. Nevertheless, other past quantitative NIR simulation studies also used the similar value of *g* (*g* = *0*) [7, 14].

**Fig. 5:**
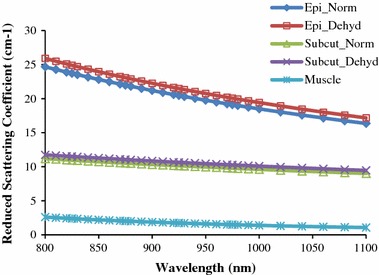
Reduced scattering coefficient spectra from the values obtained using Eq. (4). Reduced scattering coefficient spectra of normal (Norm) and dehydrated (Dehyd) epidermis–dermis (Epi), $\mu_{s}^{{{\prime }epidermis}}$ (λ) and subcutaneous fat layer (Subcut), $\mu_{s}^{{{\prime }subcutaneous}}$ (*λ*) and included reduced scattering coefficient spectra of normal hydrated muscle layer, $\mu_{s}^{{{{{\prime }muscle}} }}$ (*λ*)

A past study has shown that similar values of $\mu_{s}^{{{\prime}}}$ were obtained from measurements on Caucasian skin and African skin, thus values of the *n* and $\mu_{s}^{{{\prime}}}$ in this study were similar while *V*_*melanin*_ varied [17]. Decreasing water in skin tissue estimated to increase *n* by 5% [34], hence dehydrated tissues of epidermis as well as dehydrated subcutaneous possess 5% higher $\mu_{s}^{{{\prime}}}$. Findings by Roggan et al. from 456 to 1064 nm were used for the *n* with normal hydration, *n* ^*epidermis*^(*λ*) = 1.4, *n* ^*subcutaneous*^(*λ*) = 1.44 and *n* ^*muscle*^(*λ*) = 1.37 [35, 36]. For dehydrated tissue, the *n* ^*epidermis*^(*λ*) and *n* ^*subcutaneous*^(*λ*) became 1.47 and 1.512 respectively (values increased 5%) due to the water loss effect on scattering. An equation developed from [16] was used to define normal hydration values of scattering $\mu_{s}^{{{\prime}}}$, as;

4 $$\mu_{s}^{{\prime}} \left( \lambda \right)^{layer} = a \left( {\frac{\lambda }{500\, (nm)}} \right)^{ - b}$$

where $\mu_{s}^{{{\prime}}}$(*λ*) is the reduced scattering coefficient spectra of the given layers. Dimensionless values for *a* and *b* were assigned based on [16]: 45.3 and 1.29 respectively for *μ*_*s*_ *′* ^*epidermis*^(*λ*), 15.4 and 0.68 respectively for *μ*_*s*_ *′* ^*subcutaneous*^(*λ*) while for *μ*_*s*_ *′* ^*muscle*^(*λ*), 9.8 was set for *a* and 2.82 for *b.* Figure 5 shows normal and dehydrated (after increased by 5%) values of *μ*_*s*_ *′* ^*epidermis*^(*λ*) and *μ*_*s*_ *′* ^*subcutaneous*^(*λ*) as well as only normal hydrated values of *μ*_*s*_ *′* ^*muscle*^(*λ*). The graphs in Fig. 5 are plotted from the values obtained using Eq. (4).

A source and a detector were created in GAMOS following the specifications and parameters shown in Table 2. The source and the detector were positioned at +45° and −45° angles to the skin surface respectively with 10 mm separation. From the detector numerical aperture (*NA*) of 1.0 (in Table 2), its acceptance angle was calculated using an equation of θ = *sin* ^−*1*^ (*NA*). Fundamentally, a detector can collect light if the light falls within an angle that is two times its acceptance angle; hence, the cosine corrector detector can record photons from 0° angle up to 180° angle.

**Table 2:** Specifications and parameters of the source and the detector utilised in GAMOS simulation

Parameters/specifications	Source—SMA fibre cable	Detector—cosine corrector
Experimental equipment	Thorlabs, M28L01	Thorlabs CCSA1
Diameter of core fibre (mm)	0.4	4.0
Numerical aperture	0.39	1.0
Materials	Pure silica (core), TECS hard (cladding)	PTFE or Teflon (core)
Refractive index	1.47	1.315
Implement Geant4 Material Database function (based on core materials)	G4_SILICON DIOXIDE	G4_TEFLON
Angle to the normal skin surface	+45°	−45°

**Fig. 6:**
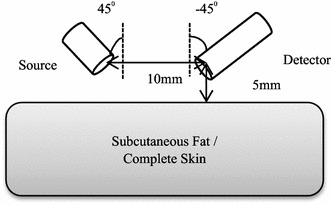
Simulation diagram of the source-detector arrangement used in the simulation

**Fig. 7:**
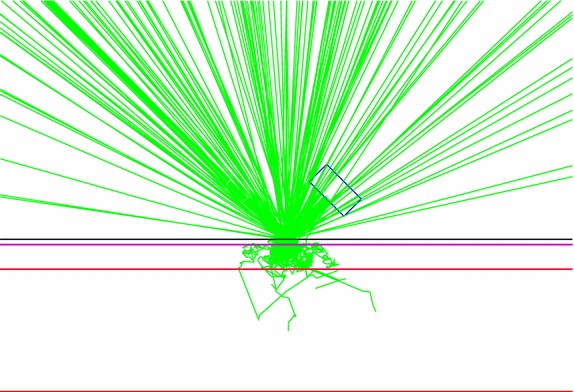
An example of the traces of photon paths during the simulation in GAMOS (2D view). The photons trace was obtained with regard to Fig. 6, where the photons were launched into the developed skin model (upper epidermis–dermis, middle subcutaneous fat, lower muscle) by the source at +45° angles to the skin surface and the escaped photons were recorded by the detector at −45° angles to the skin surface. The source and detector separation was at 10 mm

Following the development of the skin models and the source-detector arrangement, 10^8^ optical photons from 800 to 1100 nm (in 50 nm intervals) were launched by the light source. The photons were launched into the two single fat layer models and four skin models. At the receptor side, the detector recorded photons quantitatively as a function of wavelength. NIR reflection was defined to be the number of recorded photons over the number of launched photons in term of percentage. Figure 6 shows the source-detector arrangement used in the simulation and Fig. 7 demonstrates an example of the traces of photon paths during the simulation in GAMOS (2D view). The sensitivity of the response to the changing thickness of fat or the slope of reflected NIR intensity over 14 intervals of 1 mm thickness from 1 to 15 mm (expressed as percent reflection change per mm) was calculated using the following where *t* is the step index of the thickness of fat;

5 $$Sensitivity = \mathop \sum \limits_{t = 1}^{14} \left( {\frac{{ Reflection_{t + 1} - Reflection_{t} }}{14}} \right)$$

## Results

**Fig. 8:**
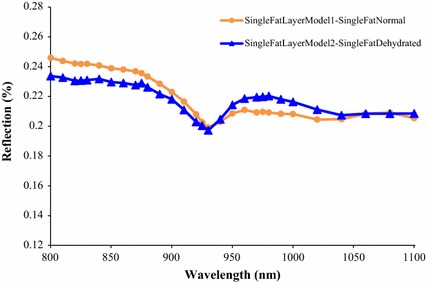
NIR reflection spectra from two developed single fat layer models at varied *V*_*water*_. The single fat model 1 was the single fat layer having normal hydration while dehydrated single fat layer was for the single fat layer model 2. The fat thickness was 5 mm

**Fig. 9:**
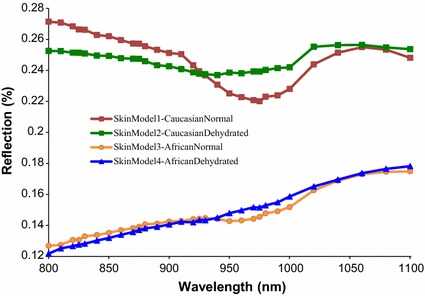
NIR reflection spectra from four developed skin models at varied *V*_*melanin*_ and *V*_*water*_. The skin model 1 and the skin model 2 were the Caucasian skins (lower *V*_*melanin*_) with normal hydration (higher *V*_*water*_) and dehydrated (lower *V*_*water*_) respectively. The skin model 3 was the African skin (higher *V*_*melanin*_) having normal hydration (higher *V*_*water*_) while the dehydrated African skin (higher *V*_*melanin*_ and lower *V*_*water*_) was for the skin model 4. The thickness of the subcutaneous was 5 mm

The effect produced by varying the *V*_*water*_ can clearly be seen in the NIR spectra from the single fat layer models in Fig. 8. The variation is minimal at ranges of wavelengths from 890 to 940 nm and also from 1010 to 1100 nm (note that the *V*_*melanin*_ was not involved because melanin only presents in the epidermal layer of the skin). Similar minimal variation at the two ranges of wavelengths (from 890 to 940 nm and also from 1010 to 1100 nm) is also seen in the spectra of complete skin models having different hydration presented in Fig. 9, where in Fig. 9, *V*_*water*_ was less for models 2 and 4. Different skin colours has a significant affect on the NIR spectra in Fig. 9 as photons were absorbed more in African skin (skin models 3 and 4) than Caucasian skin (skin models 1 and 2) due to the presence of greater melanin constituent (*V*_*melanin*_) in the African skin.

**Fig. 10:**
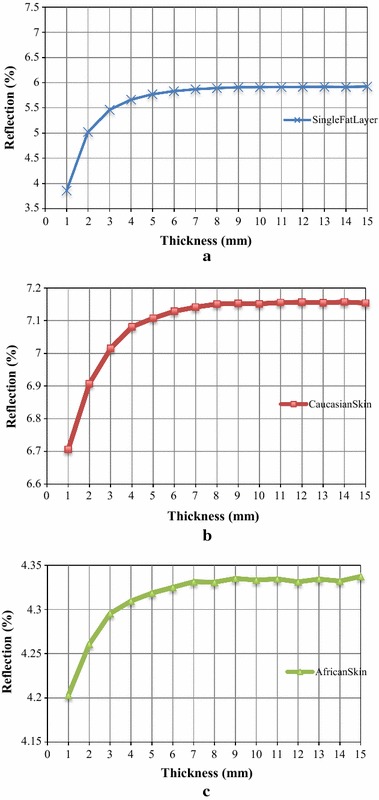
Logarithmic response of NIR reflection (%) over the thickness of fat at 930 nm. Logarithmic response from **a** the single fat layer, **b** the full complete Caucasian skin and **c** the full complete African skin

Figure 10a–c show the NIR reflection from the single fat layer, Caucasian skin and African skin respectively at 930 nm. The NIR reflection increases logarithmically with increases in the thickness of fat. Note that wavelength at 930 nm was one of the wavelengths that showed minimal variation due to changes in *V*_*water*_ over the NIR spectra (in Figs. 8, 9). The logarithmic response of NIR intensity with thickness of fat is due to the affect of scattering being dominant over the affect of absorption in the adipose tissue. More photons were increasingly being scattered back with increases of the thickness until reaching a critical thickness, where the change of the response becomes saturated and less sensitive, and there is negligible increase in backscattered light with any further increases of the thickness of fat.

The sensitivities [using Eq. (5)] were reduced with the presence of other skin layers (full complete skin), where the sensitivities were 13.1 × 10^4^%/mm for the single fat layer, 2.81 × 10^4^%/mm for the complete Caucasian skin and 0.81 × 10^4^%/mm for the complete African skin. Reflected NIR intensity obtained from African skin was lower than Caucasian skin. The different sensitivities acquired between these skin colours shows the influence of skin colours in NIRS measurements when there is no adjustment for melanin.

## Discussion

To our knowledge, this is the first study that has applied a source-detector arrangement at 45° angles to the skin surface in GAMOS simulations to study the effect of varying skin colour and hydration over NIR intensity particularly for body fat sensing for neonates. We also first studied and analysed the relationship between NIR reflectance and thickness of fat due to the differing relationships found in the past studies.

Our findings showed that varying hydration in the skin resulted minimal changes to the NIR intensity at two ranges of wavelengths from 890 to 940 nm and also from 1010 to 1100 nm. Those wavelengths (within the two ranges) may consider to be implemented in the development of a NIRS device for the measurement of body fat in order to address the wide variation of water in the neonates’ body. Total body water particularly in newborns normally fluctuates in their first week of life due to the critical transition period from foetus in womb to newborn [37]. Our study showed that increasing melanin volume in the skin decreased the sensitivity of the measurement to the intended goal of determining subcutaneous fat thickness. This limitation is expected and a considerable challenge because skin colour varies from one neonate to another. The strong influence by melanin in NIRS measurements was also found in a past study, which they studied reliability of NIRS in people with dark skin pigmentation for tissue oxygen delivery application and found that the presence of melanin clearly interfered quality of the reflected NIR signal [38]. Their NIR device failed to register tissue saturation more often in individuals with darker skin. One of the possible ways to solve this problem is to quantify the skin colour using a promising Antera 3D device and include values of the measured colour space (L*a*b*) in the developed NIR equation model of body fat [39]. Another alternative method is to include the use of ratios of reflection at different wavelengths in the developed NIR equation model of body fat [40]. Our recent studies have implemented the ratios technique in the developed body fat percentage (BF%) model from clinical measurements on neonates using NIRS referring to BF% from an air displacement plethysmography (ADP) [41]. The developed equation model consisted three ratios using five different wavelengths with a parameter of sex. The results demonstrated a significant correlation and in agreement with the ADP BF% (R-squared of 0.82 (p < 0.001) and RMSE 2.1%). However, our subjects recruited from Sydney, Australia were majority from white skin colour (n = 26) while only four subjects from dark skin. This has recently been expanded with our study of 98 infants (dark skin) from Soweto, South Africa [42]. The ratios based model combining with weight and sex yielded a correlation R-squared of 0.773 (p < 0.001) and RMSE 4.6%. The high correlation R-squared obtained from dark skin indicates that the use of ratios can reduce the sensitivity due to melanin, however other clinical studies with higher number of subjects studied are required to confirm this result. The use of reflection ratios at different wavelengths may also reduce the effect of dissimilar reflected NIR intensity values obtained from Africans and Indians even though they possessed similar content of melanin (*V*_*melanin*_), which may due to different sizes of melanosomes [43]. Larger size of melanosomes in African skin possibly increase forward scattering, thus less backscattered light(s) is captured [44]. Our obtained NIR reflection recorded by the detector exhibited small NIR reflection values and may be considered as an unamplified signal by the photo-detector at the receptor side. Thus in the final implementation, the NIR reflection values might be increased with an amplifier.

The relationship between the reflected NIR intensity and the fat thickness in the past studies showed a range of different forms those being either a logarithmic, a peak or an exponential. We obtained logarithmic response, which in agreement with curves obtained from the majority of the past studies ([12, 8] and some results of [7]). The study in [14] and one result from [7] obtained a polynomial or exponential relationship while for [11], it showed a peak curve, where light intensity increased first and then decreased at a 10 mm fat thickness. A potential explanation for these differences is the different of source-detector separations used in the studies. For example in [7], the logarithmic relationship that was applied to small source-detector separations (less than 30 mm) changed to an exponential relationship at 40 mm source-detector separation at which point it agreed with [14]. In [11] that used 8.5 mm source-detector separation, two different equation models were developed, where the second model was applied after 10 mm fat thickness. A similar logarithmic relationship to other studies can be obtained if the quantitative method was used [11]. Other reasons for the difference of the curves acquired (a logarithmic, a peak or an exponential) may also be due to the unknown differences in source diameter and angle of incidence used in the study.

The critical thickness of the logarithmic response indicates that there is a maximum thickness detection (MTD) limit for NIRS to measure body fat. The MTD defines whether or not NIRS could be used to detect and monitor body fat particularly low body fat (undernutrition). Information of the thickness of fat in real neonates was found to be 3.0 to 5.0 mm in normal full term neonates while only 1.7 to 3.0 mm in low birth weights [45]. While the past studies only showed and discussed certain aspects of the curve response [7, 8, 11, 12], to our knowledge there was no particular study defining optimal MTD. Hence, we suggest implementing a phantom experiment to test power sources emitted and types of NIR equipment used for defining an optimum cut off value (or MTD). The cut off is when the slope of the measured NIR intensity over thickness drops below a defined level, and becomes small enough and unchanged (constant) on increases of thickness.

There are several limitations in this study that need improvement and further study. The effect of the volume fraction variation over NIR spectra is expected to decrease with the introduction of a more complex skin model, where other components can contribute to the overall volume. Since we used and assumed the optical property value of anisotropy, *g* to be constant at various concentrations of the melanin and water, the effect on NIR spectra may be different with the use of actual *g* values to the respective tissue layer. However, the *g* values based on those conditions (varied melanin and water) were not available in the literature. The third limitation is that we assigned optical parameters from adult skin, thus the effect of the volume fraction variation on NIR spectra may be different in newborn skin. Total body water found in a newborn infant’s body is generally different from an adult, which is 81% in an infant while 73% in an adult [46]. For different skin colours, the difference in distributions and quantities of melanin pigments in the epidermal layer determines the range of colours of skin [47]. Presence of melanin in newborn infant’s epidermis may influence the NIRS results in a different way to the presented model with adult values. This is because it was found that the production of melanin was lower in the newborns but numbers of melanocytes (melanin pigments) per area was quantitatively comparable with the adults [48, 49]. Other factors including size of cells and size of fibre bundles in the skin tissues may also influence the NIRS results as past studies found that size of cells and fibre bundles in the neonatal skin tissue were in smaller sizes than that of the adults [50, 51].

As regards the size of cells and fibre bundles, light penetration in the neonatal skin would be expected to be less than in the adults because the smaller objects reduce forward scattering of the light or the anisotropy value, where the forward Mie scattering is dependent on size of objects [52]. Thus more backscattered light (reflected NIR intensity) would be captured. Contrarily, increasing total body water in neonates would decrease the value of refractive index and scattering coefficient [34], which leads to a decrease of the backscattered light. The behaviour of the light transport and the captured backscattered light in comparison with the adults can be utilised for parameterisation of the optical parameters in a developed model for obtaining the new NIR reflection values. However, comparing with the values of optical parameters from measurements on the real skin tissue of neonates is essential.

## Conclusions

The NIR reflection showed higher sensitivity at lower thicknesses of subcutaneous fat, which shows good potential for the use of the NIRS method for the detection of under-nutrition in newborn infants particularly in low-income settings, where newborns are at risk for under-nutrition and morbidity. Selecting correct wavelengths for measuring body fat is necessary to circumvent the influence on absorption and scattering that occur with variation of hydration and skin colour in the skin tissue. Wavelengths within a range from 890 to 940 nm and also from 1010 to 1100 nm would be the wavelengths to be considered in developing the NIRS device due to the minimal affect on reflected NIR intensity at varied *V*_*water*_. For different skin colours meanwhile, sensitivities and reflected NIR intensity decreased with increases of melanin, which indicates that NIR is sensitive to changes in melanin. Thus, solutions to overcome this limitation in developing the real NIRS device are necessary including correction by an independent measurement or by a modeling approach. The effectiveness of the suggested solutions in addressing the limitations due to hydration and skin colour would also be validated via in vitro experiment, where NIR reflection response would be tested on the developed skin tissues using an epoxy resin mimicking the real skin tissue with varied water concentration and ink (melanin) [53]. As we utilised some optical parameters obtained from adult skin in this study, further studies using equivalent parameters of newborn skin are essential, but defining first the values of the optical properties of neonates by measurements on the real neonatal tissue or by parameterisation approach in the developed model based on the results from adults is urged. As regards the size of melanosomes were different between ethnics even though they possessed similar values of *V*_*melanin*_, it also suggests further study of the effect of different hydration and skin colours on NIR spectra for other ethnics including Indian and Chinese with using their optical parameters of the skin for comparison with the Caucasians and Africans. For implementation, our next step is to develop a NIRS body fat device which can compensate for various skin colours and using multiple LEDs at several wavelengths.

## Additional files

**Additional file 1: Table S1.** Comparison of body composition methods using FOM equation and the measurements concept of each method. The variables in the FOM equation include estimated cost (including equipment set-up), estimated measurement time, requirement for skilled operators, noninvasiveness, mobility, and safety. The FOM should be the highest for the best device, which is the NIR method.

**Additional file 2: Table S2.** Literature review of fat measurements using NIRS by simulation and phantom experiment.

**Additional file 3.** Absorption coefficient spectra of water, hemoglobin and pure fat.

## Abbreviations

NIR: near infrared
NIRS: near infrared spectroscopy
MTD: maximum thickness detection
MCML: Monte Carlo multilayer
GMD: Geant4 material database
BF%: body fat percentage
