# Supplementary material for: Near infrared spectroscopy for body fat sensing in neonates: quantitative analysis by GAMOS simulations
Source: Biomed Eng Online. 2017 Jan 11;16:14. doi: 10.1186/s12938-016-0310-y (PMC5234151; doi:10.1186/s12938-016-0310-y)
Supplement: Supplementary file 1 — Additional file 1: Table S1. Comparison of body composition methods using FOM equation and the measurements concept of each method. The variables in the FOM equation include estimated cost (including equipment set-up), estimated measurement time, requirement for skilled operators, noninvasiveness, mobility, and safety. The FOM should be the highest for the best device, which is the NIR method. [file 12938_2016_310_MOESM1_ESM.pdf]

- File name: Additional file 1
- File format: .pdf
- Title of data: Table S1. Comparison of body composition methods using FOM equation and the measurements concept of each method.
- Description of data: Comparison of body composition methods using FOM equation and the measurements concept of each method. The variables in the FOM equation include estimated cost (including equipment set-up), estimated measurement time, requirement for skilled operators, noninvasiveness, mobility, and safety. The FOM should be the highest for the best device, which is NIR method (bolded).

**Methods**	**Primary Measurements**	**Estimated Cost (including equipment set-up)**	**Estimated Measurement Time**	**Requirement for Skilled Operator**	**Non-invasive**	**Portable**	**Safety**	**Figure of Merit, FOM (FOM= skilled operator + noninvasive + portable + safe – cost - measurement time)**
Skinfold thickness (SFT)	Regional adipose tissue (subcutaneous fat)	Low	20 s	High. Due to high variability	Yes	Yes	High safety. Does not use ionising radiation.	5
Near infrared interactance (NIR)	***Regional adipose tissue (subcutaneous fat)***	***Low***	***10 minutes***	***Low***	***Yes***	***Yes***	***High safety. Does not use ionising radiation.***	***6***
Ultrasound	Regional adipose tissue (subcutaneous fat) and lean muscle mass	High	20 minutes	High. For handling the equipment	Yes	No	High safety. Does not use ionising radiation.	1
Computer Tomography (CT)	Total body volume, specific regional muscle and bone density	High	20 minutes	High. For handling the equipment	Yes	No	Not encouraged for newborns. Ionising radiation. Effective dose from 5.4μSv for the total body scan [54].	0
Dual-energy X-ray absorptiometry	Total body water (TBW), lean mass and regional	High	20 minutes	High. For handling the	Yes	No	Not encouraged for newborns. Effective dose	0

(DEXA)	body fat, bone mineral content.			equipment			from 1.1mSv for the whole body [55].	
Stable Isotope Dilution (SID)	TBW and extracellular fluid	High	20 minutes	High. For accurate dose sampling and processing	No	Yes	High safety. Does not use ionising radiation.	1
Air-displacement plethysmography (ADP)	Total body volume and total body fat	High	10 minutes	Medium. For handling the equipment	Yes	No	High safety. Does not use ionising radiation.	3
Magnetic resonance imaging (MRI)	Total and regional adipose tissue (subcutaneous, visceral and inter-muscular)	High	20 minutes	High. For handling the equipment	Yes	No	Not encouraged for newborns. The effect of high magnetic fields are unknown. Does not use ionising radiation.	0

The variables in the FOM equation include estimated cost (including equipment set-up), estimated measurement time, requirement for skilled operators, noninvasiveness, mobility, and safety. The FOM should be the highest for the best device, which is the NIR method. Cost is low=1, high = 2; measurement time is low = 1 if <1 minutes, medium = 2 if 2 minutes to 10 minutes, high =3 if >20 minutes; skilled required is low=3, medium = 2, high = 1; non-invasive is Yes=2, No= 1; portable is Yes=2, No= 1; safe is = 1 if radiation, high safety = 2.
