# Supplementary material for: Near infrared spectroscopy for body fat sensing in neonates: quantitative analysis by GAMOS simulations
Source: Biomed Eng Online. 2017 Jan 11;16:14. doi: 10.1186/s12938-016-0310-y (PMC5234151; doi:10.1186/s12938-016-0310-y)
Supplement: Supplementary file 2 — Additional file 2: Table S2. Literature review of fat measurements using NIRS by simulation and phantom experiment. [file 12938_2016_310_MOESM2_ESM.docx]

- File name: Additional file 2
- File format: .docx
- Title of data: Table S2. Literature review of fat measurement using NIRS by simulation and phantom experiment
- Description of data: The table below is the literature review of fat measurement by NIRS

| **No** | **Wavelengths** | **Applications/ Populations** | **Objectives/ Aims** | **Simulation** | **Phantom Experiment** | **Source-detector Arrangement/**  **Type of Transmission** | **Layers** | **Curves Response** | **Other Findings** |
| --- | --- | --- | --- | --- | --- | --- | --- | --- | --- |
| 1 | 760nm  [5] | Obesity monitoring in adults | Studied NIR response on changes of fat thickness | Monte Carlo Multilayer (MCML)   - Thickness of fat: 1mm to 10mm in 1 mm interval. - Source-detector separation: 0mm to 40mm in 10mm interval. | - | 90-degree / Reflectance | Fat-muscle layer & Complete skin | At 10mm, 20mm, 30 mm source-detector = logarithmic.  Curve of graph at 40mm source-detector = exponential. | 1. Intensity of photon reflection increased at closer distance of the source-detector. |
| 2 | 770nm  [6] | Obesity monitoring in adults | 1. Studied performance of fat measurement by NIRS using different types of source-detector pair. 2. Validation of MCML with in-vitro phantom experiment. | Monte Carlo Multilayer (MCML)   - Thickness of fat: 5mm to 30mm in 5mm interval. - Source-detector separation: 10mm, 15mm, 20mm and 25mm | Gelatine as based material.  Devices: A silicon detector with 2 different sources; 5 lamp LEDs (view angle 20-degree) OR multiple miniaturized chips LED (view angle 120 degree).   - Thickness and source-detector separation: similar as in the simulation. | 90-degree / Reflectance | Fat-muscle layer | Logarithmic | 1. Intensity of photon reflection increased at closer distance of the source-detector. |
| 3 | 770nm  [7] | Obesity monitoring in adults | Studied on light path in the skin at varied source-detector separation | Monte Carlo Multilayer (MCML)   - Source-detector separation: 3mm, 5mm, 10mm and 20mm | - | 90-degree / Reflectance | Complete skin | - | 1. Simulation – Source-detector at 10mm and 20mm had higher sensitivity to changes of fat thickness than source-detector at 3mm and 5mm. |
| 4 | 633nm and 900nm  [8] | Obesity monitoring in adults | Studied optical photon distribution in subcutaneous tissue of a complete skin model. | Monte Carlo Multilayer (MCML) | - | 90-degree / Reflectance | Complete skin |  | 1. Distributed photon absorption in the fat tissue at 633nm was lower that at 900nm, due to effects of melanin and hemoglobin in epidermis and dermis layer. |
| 5 | 1300nm  [9] | Nutritional status monitoring for adults, postoperative evaluation of liposuction. | 1. Define agreement of NIR ex-vivo and in-vivo measurements. 2. Develop NIR equation models of fat thickness measurements. | - | 1. Used devices– Developed devices consisting of 3 LEDs and 2 InGaAs photo diodes 2. Subjects - Ex-vivo (pork skin), in-vivo (human). 3. 2 equations were developed; a 3-layered and a 2-layered equation models. The 2-layers equation was applied after a critical thickness.  - Thickness of pork fat: 0mm to 20mm in 2mm interval. - Source-detector separation: 8.5mm | 90-degree / Reflectance | Complete skin | Increased and then decreased at a critical thickness | 1. NIR from phantom measurements agreed with NIR from human fat measurements. |
| 6 | 600nm-1000nm  [10] | Nutritional status monitoring for neonates | 1. Define optimum wavelengths and sensitivity of source-detector separation. 2. Effect of hydration and melanin | GAMOS simulation   - Source-detector separation: 15mm to 29mm in 1mm interval - Thickness of fat: 1mm to 10mm in 1mm interval | - | 90-degree / Reflectance | Complete skin | Logarithmic | 1. Wavelength range at 630nm to 690 nm was ideal for fat measurement at 26 to 29 mm distance of the source-detector. 2. The selected wavelength range was not ideal to define different hydration level. |
| 7 | 730nm  [11] | Liposuction surgery. | Validation between simulations and phantom measurements with considering characteristics of LED in simulation | Monte Carlo Multilayer (MCML) | Liquid based (intralipid).  NIRS device – 6 LEDs and a photo detector (Ø1.2mm). | 90-degree / Reflectance | Single fat layer | - | 1. Obtained correlation coefficient of 0.977 between simulation and phantom experiment. |
| 8 | 760nm  [12] | Influence of subcutaneous fat layer on sensitivity of muscle-oxygenation by NIRS | Validation of MCML with in-vitro phantom experiment. | Monte Carlo Multilayer (MCML)   - Source-detector separation: 40mm - Thickness of fat: 2mm-12mm, 2 mm interval | Agar as base material.  Devices: a halogen light source and a spectrometer as detector   - Source-detector separation and thickness of fat were similar as simulations | 90-degree / Reflectance | Fat-muscle layer | Polynomial relationship (exponential curve) | The MCML simulations agreed with the experiments. |
